# Atomistic Molecular Dynamics Simulations of Lipids Near TiO_2_ Nanosurfaces

**DOI:** 10.1021/acs.jpcb.1c04547

**Published:** 2021-07-16

**Authors:** Mikhail Ivanov, Alexander P. Lyubartsev

**Affiliations:** Department of Materials and Environmental Chemistry, Stockholm University, SE-106 91 Stockholm, Sweden

## Abstract

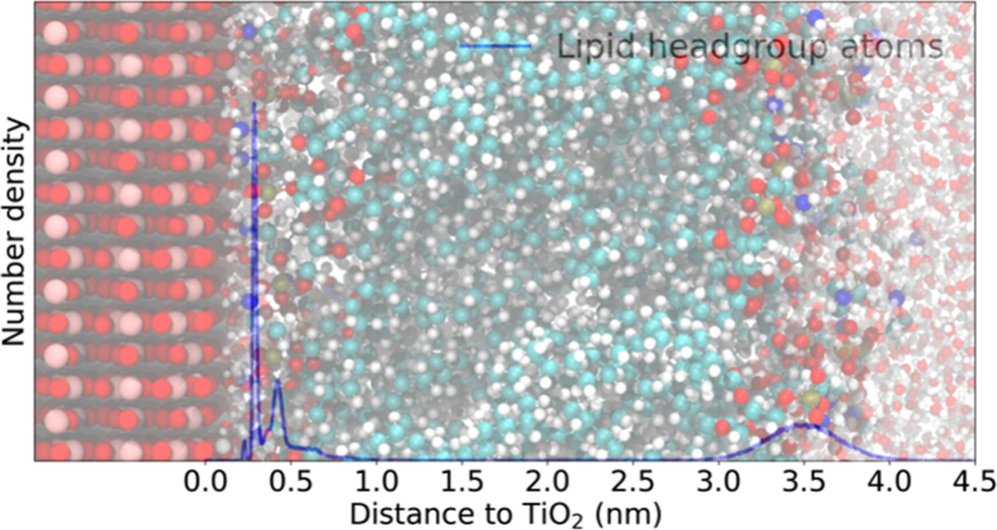

Understanding of interactions between inorganic nanomaterials and biomolecules, and particularly lipid bilayers, is crucial in many biotechnological and biomedical applications, as well as for the evaluation of possible toxic effects caused by nanoparticles. Here, we present a molecular dynamics study of adsorption of two important constituents of the cell membranes, 1,2-dimyristoyl-*sn*-glycero-3-phosphocholine (DMPC) and 1-palmitoyl-2-oleoyl-*sn*-glycero-3-phosphoethanolamine (POPE), lipids to a number of titanium dioxide planar surfaces, and a spherical nanoparticle under physiological conditions. By constructing the number density profiles of the lipid headgroup atoms, we have identified several possible binding modes and calculated their relative prevalence in the simulated systems. Our estimates of the adsorption strength, based on the total fraction of adsorbed lipids, show that POPE binds to the selected titanium dioxide surfaces stronger than DMPC, due to the ethanolamine group forming hydrogen bonds with the surface. Moreover, while POPE shows a clear preference toward anatase surfaces over rutile, DMPC has a particularly high affinity to rutile(101) and a lower affinity to other surfaces. Finally, we study how lipid concentration, addition of cholesterol, as well as titanium dioxide surface curvature may affect overall adsorption.

## Introduction

Titanium dioxide nanoparticles (NPs) are ubiquitous in personal care products,^1,2^ food,^3−5^ various paint and self-cleaning coatings,^6−10^ as well as in advanced applications like photocatalysts and dye-sensitized solar cells.^11−15^ Furthermore, TiO_2_ is used as a substrate for solid-supported phospholipid bilayers for biosensor applications.^16−19^ However, recent studies have raised concern about potential health risks of TiO_2_ NPs associated with their toxicity.^4,20−25^ TiO_2_ is known for producing reactive oxygen species, which can damage neurons,^26^ oxidize, and rupture cell membranes.^27,28^ Exposure to TiO_2_ NPs can cause lung inflammation and increased blood coagulation connected with cardiovascular diseases.^23,24^ Although many possible adverse outcomes of TiO_2_ NPs exposure are known, the molecular mechanisms of the nanotoxicity are uncertain.^29,30^ To study the nanotoxicity mechanisms, a large number of experimental studies on model systems were carried out.^19,25,27,31−37^ It is well accepted that in an organism, a nanoparticle becomes covered by a layer of proteins, lipids, and other organic molecules,^38^ which is called protein corona and which determines, in a large extent, the further fate of NP in the organism and potential toxic effects. Coreas et al.^32^ have studied protein corona formation of TiO_2_ NPs in simulated gastrointestinal digestion and shown that lipids dominate the biocorona. Runa et al.^27^ have reported that TiO_2_ NPs bind to the cell surface and oxidize the lipids of the plasma membrane. Another study has shown that TiO_2_ NPs can penetrate layers of dipalmitoylphosphatidylcholine (DPPC) lipids.^37^ More detailed information about the interactions of lipid molecules with the inorganic surfaces is reported by Yu et al.^25^ They have studied the toxicity of anatase and rutile NPs (20–40 nm) and have found that anatase NPs have a higher affinity toward proteins and mainly impair mitochondrial function. On the other hand, rutile NPs have a higher affinity toward an important plasma membrane phospholipid—phosphatidylethanolamine (PE). Other studies have reported that the phosphate group of phosphatidylcholine (PC) lipids can bind to the surface of metal oxides and the formation of supported lipid bilayers on TiO_2_ NPs is possible.^31,39^ However, the stability of PC bilayers is relatively low compared to silica NPs because the adsorption is based mainly on weak van der Waals interactions as bulky choline group blocks the phosphate.^35^ Wang et al.^35^ have demonstrated the importance of phosphate binding by showing that inverse PC lipids with the phosphate in the front form supported bilayers with significantly higher stability than PC bilayers on silica. IR spectroscopic study^34^ of dipalmitoylphosphatidylcholine (DPPC) lipids on TiO_2_ suggested that each choline headgroup ^+^N(CH_3_)_3_ interacts laterally with the negatively charged PO_2_^–^ of the adjacent lipid molecule.

An alternative way to study the interactions of inorganic surfaces and phospholipid membranes is by molecular simulations, which provide an atomistic insight where the role of each component can be followed.^29,30,40,41^ One of the early simulation studies of phospholipids adsorption on TiO_2_ surfaces by Fortunelli and Monti have found that the adsorption strength is strictly connected to the nature of both the lipid and the surface.^42^ The authors have pointed out that direct coordination of phosphate or carbonyl oxygens of the lipid headgroup is associated with stronger adsorption and reduced dynamics. More recently, Schneemilch and Quirke have calculated the adhesion strength of 1,2-dimyristoyl-*sn*-glycero-3-phosphocholine (DMPC) bilayers to a number of low-energy titanium dioxide surfaces.^29^ Their observations suggest that rutile surfaces have a slightly higher affinity toward the lipids than the anatase surfaces; however, both are lower compared to adhesion to amorphous silica. Lin et al.^40^ have simulated adsorption of a hydrophilic NP on a solid-supported DPPC bilayer using coarse-grained molecular dynamics (CGMD). They have reported that the adsorption behavior is largely dominated by the surface charge properties of the NP. In another computational study,^30^ a CGMD simulation of a small negatively charged NP in contact with DMPC lipid bilayer and a human serum albumin (HSA) molecule has been carried out. The simulations have shown that the NP, while coated with HSA cannot penetrate the lipid bilayer as much as the free NP, which may correspond to lower biological activity of the coated NP.

In this work, we study interactions of the two most abundant lipids in the plasma membrane, phosphatidylcholine (presented by DMPC, or 1,2-dimyristoyl-*sn*-glycero-3-phosphocholine lipid) and phosphatidylethanolamine (presented by POPE, or 1-palmitoyl-2-oleoyl-*sn*-glycero-3-phosphoethanolamine lipid) with several low-energy anatase and rutile planar surfaces and a spherical anatase nanoparticle using all-atom molecular dynamics simulations. We identify different TiO_2_–lipid binding modes as well as quantitatively characterize the adsorption and dynamics of lipid aggregates on TiO_2_ surfaces. Besides giving detailed atomistic insight into the lipid–TiO_2_ interface, the atomistic trajectories obtained in this study can be further used to construct coarse-grained models of TiO_2_ NPs and lipids for simulations of lipid–NP aggregates on larger space and time scales.^43,44^

## Methods

### System Composition

**Figure 1:**
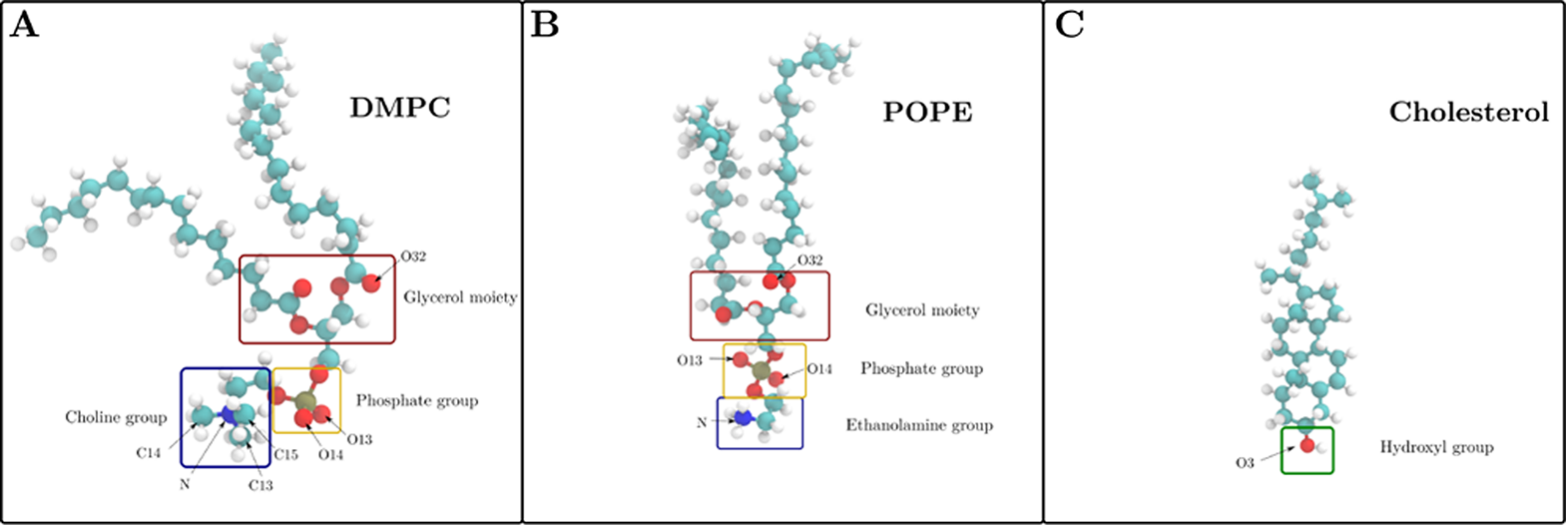
Studied lipid molecules: (A) DMPC (14:0-14:0 PC lipid), (B) POPE (16:0-18:1(*n* – 9)PE lipid), and (C) cholesterol molecule.

**Figure 2:**
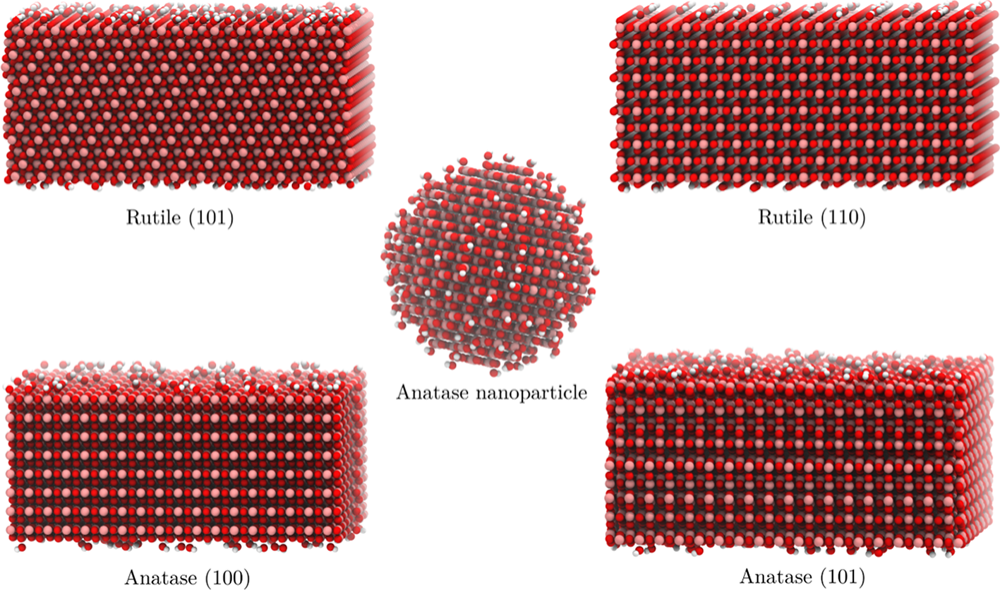
Studied TiO_2_ surfaces (side view) and nanoparticle.

Atomistic molecular dynamics simulations have been carried out for DMPC and POPE lipids in water near several TiO_2_ nanosurfaces with low surface energy:^45^ anatase(101), anatase(100), rutile(110), and rutile(101), as well as near small spherical anatase nanoparticle. In addition, the influence of cholesterol on lipid adsorption in 1:5 mixtures with DMPC and POPE lipids near the anatase(101) surface was investigated. Table 1 shows the sizes and the structures of the simulated TiO_2_ nanosurfaces. Note that the reported numbers of undercoordinated Ti atoms correspond to the TiO_2_ nanosurfaces before addition of hydroxyl groups. Details on simulated system composition and sizes are given in Table 2. The number of lipid molecules is selected in such a way that about a half of the TiO_2_ nanosurface would be occupied, assuming bilayer arrangement, adsorption of the bilayer via the headgroups, and an area per lipid equal to 0.6 nm^2^. Additional simulations of anatase(101) surface were carried out with increased number of lipids. Figure 1 shows the images of the simulated lipids. Specific parts of the polar lipid headgroups along with several atom names referred further in the text are highlighted. Studied TiO_2_ surfaces and nanoparticles are shown in Figure 2.

**Table 1:** Size and Composition of the Simulated TiO_2_ Nanosurfaces^a^

TiO_2_ nanosurface	size (Å)	*N*_Ti_	*N*_Ti(5)_	*N*_Ti(4)_	*N*_OH_	surface charge density (e/nm^2^)
anatase(101)	68.2 × 71.8 × 31.1	4536	504	0	151	–0.62
anatase(100)	68.2 × 66.7 × 28.4	4032	504	0	151	–0.67
rutile(110)	71.0 × 71.5 × 31.8	5280	528	0	158	–0.63
rutile(101)	68.9 × 71.0 × 31.4	5070	780	0	234	–0.97
anatase NP	*R* = 20 Å	921	162	96	144	–0.99

a *N*_Ti_ is the total number of Ti atoms, and *N*_Ti(5)_ and *N*_Ti(4)_ are the number of undercoordinated Ti atoms with, respectively, five and four bound oxygens before addition of *N*_OH_ hydroxyl groups.

**Table 2:** Composition of the Simulated Systems

TiO_2_ nanosurface	lipids	*N*_H_2_O_	ions	simulation box vectors, (nm)	simulation time (μs)
anatase(101)	82 POPE	11 785	94Na^+^ + 33Cl^–^	7.08 × 6.94 × 12.45	1 + 5
anatase(101)	82 DMPC	12 042	94Na^+^ + 33Cl^–^	7.08 × 6.94 × 12.43	1
anatase(100)	76 POPE	11 186	92Na^+^ + 31Cl^–^	7.08 × 6.37 × 12.62	1
anatase(100)	76 DMPC	11 313	92Na^+^ + 31Cl^–^	7.08 × 6.37 × 12.52	1
rutile(110)	85 POPE	12 025	97Na^+^ + 33Cl^–^	7.14 × 7.29 × 12.20	1
rutile(110)	85 DMPC	12 245	98Na^+^ + 34Cl^–^	7.14 × 7.29 × 12.17	1
rutile(101)	82 POPE	11 414	127Na^+^ + 32Cl^–^	7.04 × 7.22 × 11.87	1
rutile(101)	82 DMPC	11 642	127Na^+^ + 32Cl^–^	7.04 × 7.22 × 11.83	1
anatase(101)	120 POPE	11 800	94Na^+^ + 33Cl^–^	7.08 × 6.94 × 13.36	1
anatase(101)	120 DMPC	12 145	95Na^+^ + 34Cl^–^	7.08 × 6.94 × 13.32	1
anatase NP	83 POPE	93 391	309Na^+^ + 259Cl^–^	14.39 × 14.39 × 14.39	1
anatase(101)	82 POPE + 16 cholesterol	11 472	93Na^+^ + 32Cl^–^	7.08 × 6.94 × 12.50	1
anatase(101)	82 DMPC + 16 cholesterol	11 647	93Na^+^ + 32Cl^–^	7.08 × 6.94 × 12.41	1

According to experimental studies,^46−48^ at neutral pH, hydrated TiO_2_ surfaces are covered with hydroxyl groups and are negatively charged. Recent ab initio MD simulations^49^ also showed that Ti atoms exposed to water bind hydroxyl groups resulted from splitting of water molecules. The amount of hydroxyl groups depends on pH and type of the surface. Potentiometric studies of the rutile–water interface^46^ show that under standard conditions, neutral pH, and in 0.3 M NaCl, the surface charge density is about −0.62 e/nm^2^. Likewise, TiO_2_ nanoparticles consisting of anatase (80%) and rutile (20%) have a surface charge density of −0.56 e/nm^2^ under similar conditions.^47^ In our model, we bind hydroxyl groups to 5-fold-coordinated Ti atoms so that the surface charge density is close to the experimental value at neutral pH. Thus, we add hydroxyl groups to 30% of randomly picked 5-fold-coordinated Ti atoms. Additionally, we add one hydroxyl group to every 4-fold-coordinated Ti atom due to their high reactivity. The resulting surface charge densities of rutile(110), anatase(101), and anatase(100) are close to the potentiometric data; however, rutile(101) and anatase NP have more negative surface charge density due to a larger number of surface defects.

The TiO_2_ slab is placed in the middle of the simulation box with three-dimensional (3D) periodic boundary conditions. The box size in *X* and *Y* directions is defined by the slab length and width so that the slab is periodic in those directions. Initial box height is set to 130 Å (140 Å for systems with a higher number of lipids) to accommodate the TiO_2_ slab (thickness, 28.4–31.8 Å), possible formation of the lipid bilayer on both sides (2 × 40 Å) and their hydration layers (2 × 10 Å). Phospholipids (POPE, DMPC) in 76–120 molecules, and in some simulations cholesterol molecules, are inserted at random unoccupied positions in random orientations. If the subsequent simulation resulted in lipids attached to both sides of the slab, we have restarted the simulation with the lipids inserted only to the upper part of the box (with the Z axis pointing upward). After that, the box is filled with water molecules. Then, a small number of water molecules are picked at random and are substituted with Na^+^ and Cl^–^ ions to balance the negative surface charge of the slab and provide NaCl concentration of 0.15 M in the water phase of the simulated system.

Anatase nanoparticle is placed in the center of the cubic periodic box with a size of 144.4 Å. A total of 83 POPE lipids are inserted at random unoccupied positions within 15 Å from the nanoparticle surface. Then, the box is filled with water and ions are added in the same way as for the systems with the TiO_2_ slabs.

### Force Field

Lipids are described by the Slipids force field.^50,51^ For TiO_2_, we used a newly developed force field^52^ with parameters based on the analysis of electron density and water–TiO_2_ surface coordination obtained in *ab initio* simulations of the TiO_2_–water interface.^49^ In this force field, neighboring Ti and O atoms are bound by harmonic bonds so that the overall structure of the TiO_2_ sample remains fixed, but minor local motions of atoms are allowed. All force-field parameters are listed in Tables S1 and S2 in the Supporting Information. The same TiO_2_ parameters in combination with Slipids force field were employed in recent studies.^23,29^ Water molecules are represented by the TIP3P model,^53^ and for Na^+^ and Cl^–^ ions, Yoo and Aksimentiev ion parameters are used.^54^ Lorentz–Berthelot rules are applied to determine the Lennard-Jones parameters for cross-interactions.

### Simulation Protocol

For each simulated system, after the preparation of the initial state, energy minimization using the steepest gradient descent method is performed, followed by a short 100 ps pre-equilibration run at constant volume at temperature *T* = 303 K. After that, the pressure in the system is equilibrated to 1 bar using anisotropic Berendsen barostat^55^ (isotropic for the system with anatase NP) with a relaxation time of 5 or 10 ps. Pressure equilibration is run for 50 ns for anatase NP system, 100 ns for TiO_2_ slabs with a large number of lipids (120 molecules), and 10 ns for all other systems (TiO_2_ slabs with 76–82 lipid molecules). The equilibration is followed by 1 μs production run in the NVT ensemble. An additional 5 μs NVT run is performed for the anatase(101)–POPE system to assess the same properties on a longer time scale (see the Effect of Simulation Length section in the Supporting Information). Leap-frog algorithm with time step 1 fs is used to integrate equations of motion. Center-of-mass motion is removed every 100 steps. Verlet cutoff scheme^56^ with a buffer tolerance of 0.005 kJ·mol^–1^·ps^–1^ per atom is used to generate the pair lists. A minimum cutoff of 1.4 nm is used for both short-ranged electrostatic and van der Waals (vdW) interactions. Long-range electrostatics are treated using particle-mesh Ewald (PME) method^57^ with a grid spacing of 0.12 nm and cubic interpolation. Long-range dispersion corrections are applied to both energy and pressure. A velocity rescaling thermostat^58^ is used to control the temperature, which is set to 303 K with a relaxation time of 1 ps. All bonds with hydrogen atoms are constrained using the LINCS algorithm.^59^ Atom coordinates and energies are saved every 5 ps. All simulations are performed by the Gromacs 2019 software package.^60^ Visualization of the simulations is done with VMD.^61^

## Results

### General Description of Lipid Adsorption on TiO_2_ Nanosurfaces

**Figure 3:**
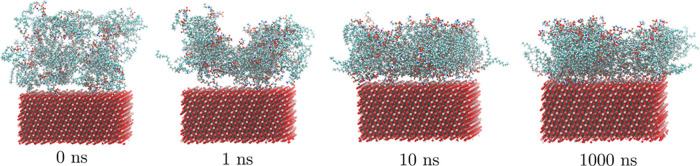
POPE lipid deposition on the anatase(101) surface.

We have observed adsorption of lipids on TiO_2_ nanosurfaces in all of the simulations. After 10–100 ns from the start of the simulations, lipids attach to the surface via the headgroups forming different aggregates. The process is illustrated in Figure 3.

**Figure 4:**
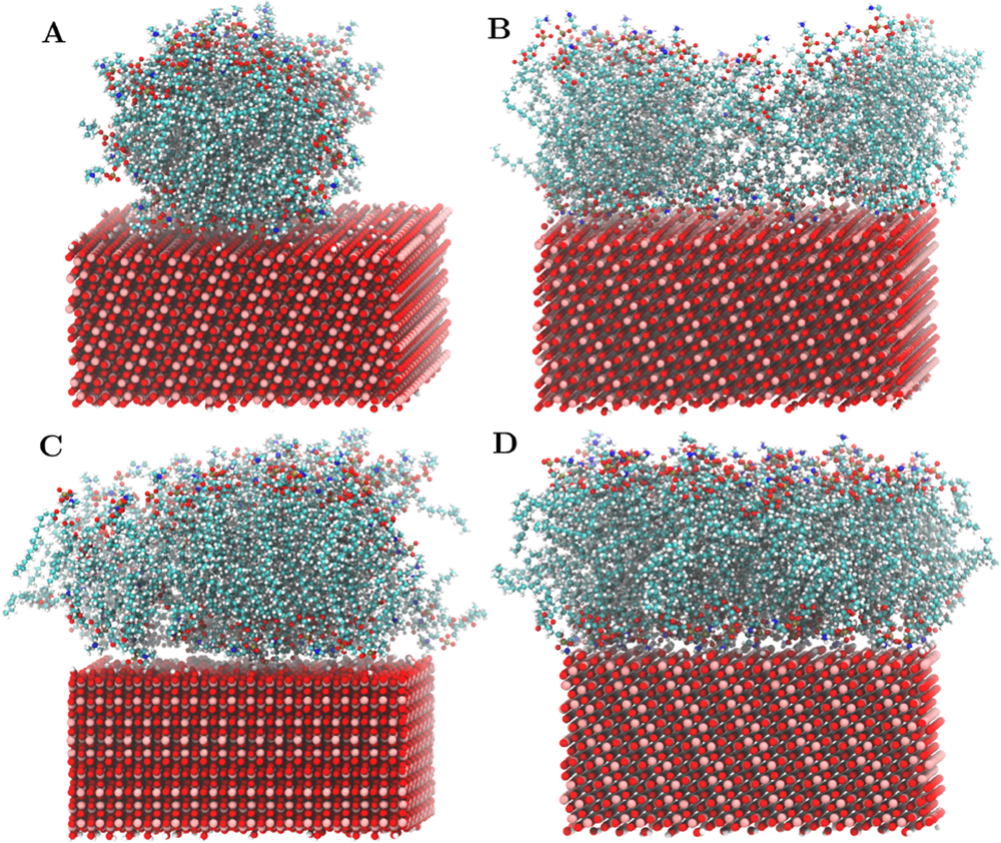
Different lipid aggregates on the anatase(101) surface. (A) DMPC cylindrical micelle (82 molecules), (B) POPE partial bilayer (82 molecules), (C) DMPC bilayer (120 molecules), and (D) POPE bilayer (120 molecules).

We found that POPE lipids tend to form partial bilayers (a bilayer with a hole) on the surface of TiO_2_. DMPC lipids, on the other hand, prefer assemblies close to cylindrical aggregates, attached to TiO_2_ surface, but with less contact compared to POPE lipids (see the snapshots of the simulated systems in Figures S1 and S2 in the Supporting Information). However, at higher lipid concentrations, DMPC lipids can also form partial bilayers on TiO_2_ surfaces, as seen from the simulation of 120 DMPC lipids near the anatase(101) surface (see the Effect of Lipid Concentrationsection in the Supporting Information). Simulations of lipids with cholesterol have shown that the cholesterol does not affect the lipid adsorption on TiO_2_ greatly and it mostly resides in the nonpolar part of the lipid aggregates. An illustration of different lipid aggregates on the surface is shown in Figure 4.

**Figure 5:**
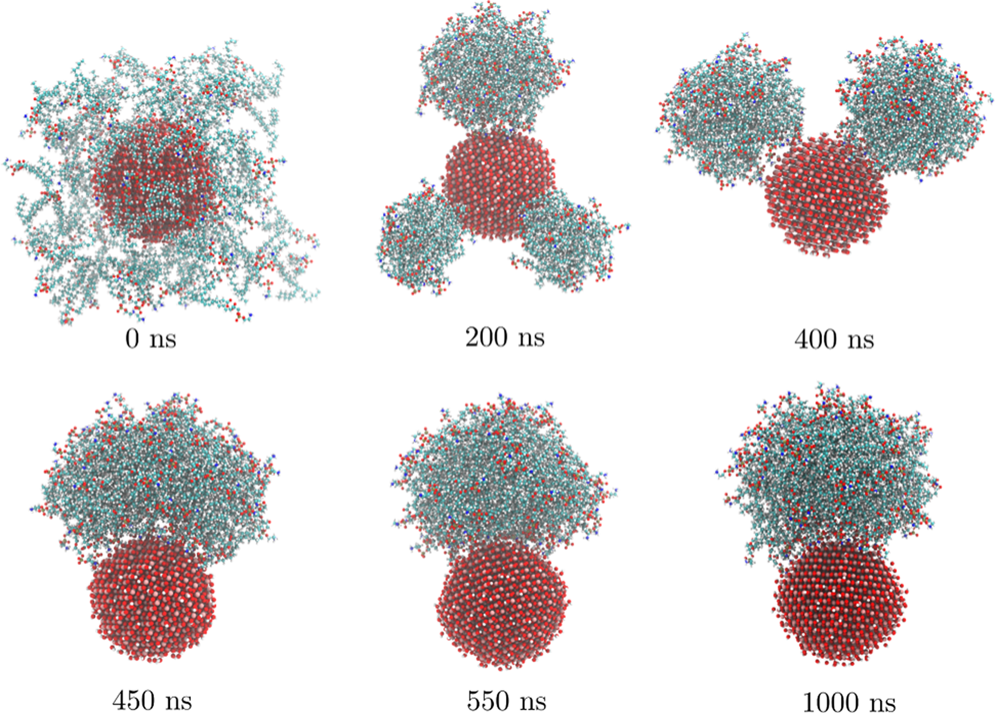
POPE lipid deposition on the anatase nanoparticle.

A more complicated phospholipid deposition process is observed for anatase nanoparticle, as seen in Figure 5. Within the first 200 ns of the simulation of anatase nanoparticle with POPE lipids, three individual spherical lipid clusters are formed at different sides of the NP. During the next 200–250 ns, the clusters migrate over the surface and merge into a single cluster. After that, the cluster becomes more spherical and the contact area with the nanoparticle decreases. The system remains stable through the rest of simulation. The last 500 ns of this simulation is used for the analysis.

### Number Density Profiles and Lipid Residence Times

**Figure 6:**
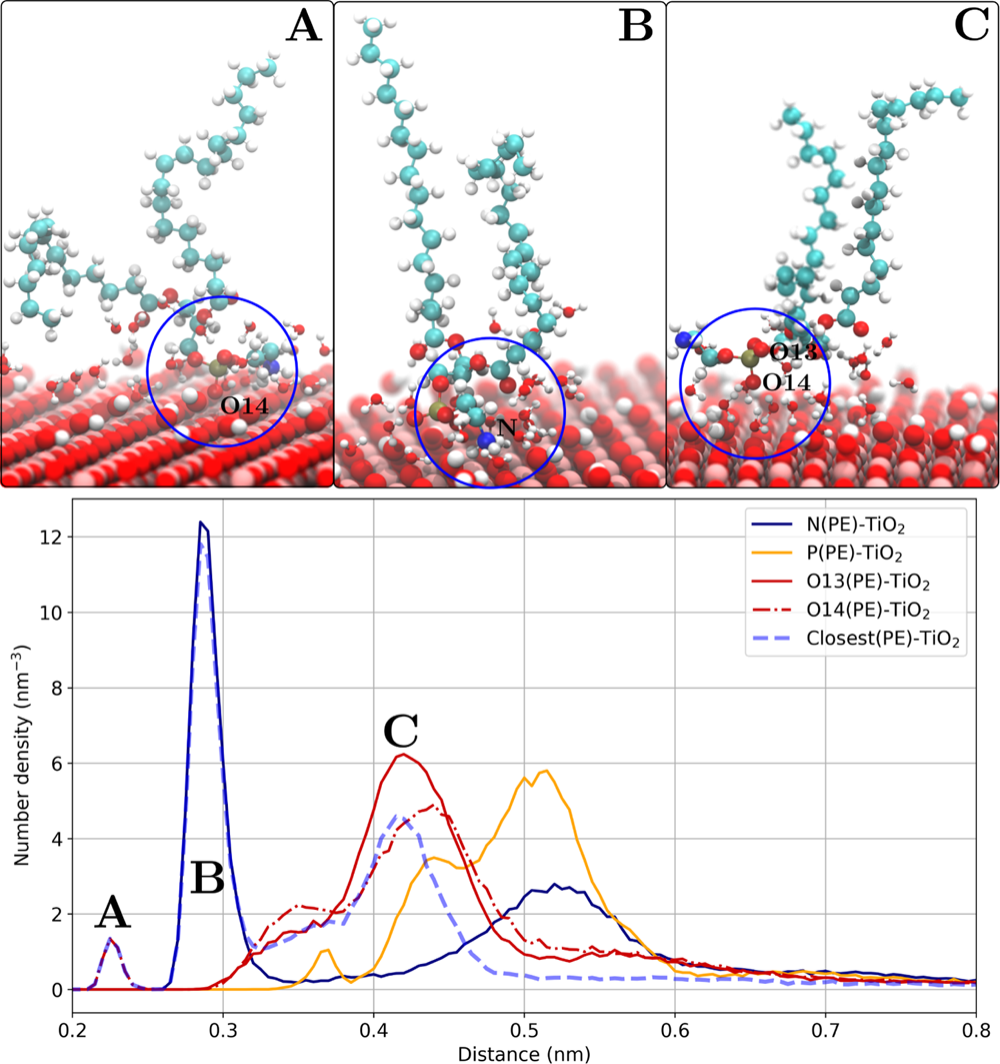
Anatase(101) slab–POPE lipid headgroup atoms number density profiles and the corresponding representations of the binding modes. (A) Phosphate group (direct contact), (B) ethanolamine group, and (C) phosphate group (water-mediated).

To characterize lipid-binding modes and compare the adsorption strength for different types of TiO_2_ surfaces and lipids, we have computed number density profiles for certain lipid headgroup atoms. The profiles are constructed from histograms of distances from each lipid headgroup atom to the nearest atom of the surface excluding hydroxyl groups. The profile is calculated for every 100 recorded frames, averaged over all number of lipid molecules and frames. The probability density values are normalized with respect to the number of frames and the bin volume. However, in the case of the number density profiles for the system with anatase nanoparticle, only the number of frames is used for the normalization. Additionally, the number density profiles of the closest lipid headgroup atom to the TiO_2_ surface are constructed to identify the possible lipid–TiO_2_ binding modes (Figure 6).

One can follow the dashed blue line (number density of the closest lipid headgroup atoms) in Figure 6 to distinguish different headgroup atoms that are directly contributing to the binding to the surface. For example, the first density peak coincides with O14 density (one of the oxygen atoms within the phosphate group) with the maximum at around 0.22 nm. It follows that some fraction of lipids binds to the TiO_2_ surface directly by the phosphate groups. The next peak with the maximum at around 0.29 nm is attributed to the large number density of nitrogen atoms from the ethanolamine group of POPE lipids. This density peak might be explained by the presence of hydrogen bonds between the inorganic surface and the ethanolamine groups of POPE lipids (corresponding radial distribution functions are shown in Figure S3 in the Supporting Information). Finally, the last number density peak of the closest atoms (there are no other heavy atoms that are closer to the surface than the selected ones) around 0.42 nm corresponds to a water-mediated interaction of the phosphate group with the surface (see Figures S4–S7 in the Supporting Information for more number density profiles).

By normalizing the number density of the closest lipid headgroup atoms, *n*(*r*), one can estimate the probability, *P*_b_, of a lipid being adsorbed through a particular binding mode
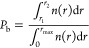
where *r*_1_ and *r*_2_ are the left and right number density peak boundaries, respectively, and *r*_max_ is the maximum distance from the TiO_2_ surface. Integration of the number density peaks for the anatase(101)–POPE system reveals that 1.2% of lipids are bound to the surface through a direct contact of the phosphate group, 18.2% through the ethanolamine group, and 21.0% through the water-mediated phosphate group contact. Overall, 46.5% of lipids approach the surface within 1 nm (an approximate length of the headgroup). This means that almost the whole lower leaflet is adsorbed, and the other half has their headgroups pointing outward.

**Figure 7:**
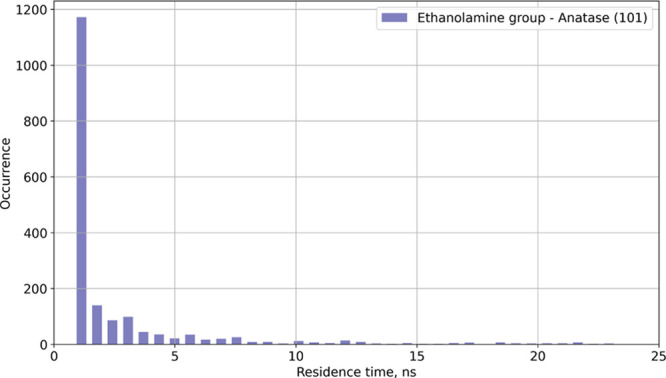
N–TiO_2_ residence time histogram for POPE lipids at the anatase(101) surface.

Another way to quantitatively describe lipid adsorption is to estimate a residence time for every resolved binding mode. This can be done by calculating the time that lipid molecule spends within a certain distance from the surface. The distance range of a binding mode is determined from the number density profile. The mean residence time is then calculated as a weighted average of the measured residence times with the occurrence as weights. An example of a residence time histogram is shown in Figure 7. Since the MD simulations are time-limited, which excludes observations of binding time longer than the time of the production part of the simulation, only the lower bound of the mean residence time can be estimated. Using the number density peak integration as well as the information about the residence times, one can determine which of the binding modes are the most prevalent and how strong they are. The analysis is performed with the help of MDtraj Python library.^62^

### Lipid–TiO_2_ Binding Modes

The following section describes different lipid–TiO_2_ binding modes that were identified in our simulated systems. Number density profiles, molecular representations, and the residence times are provided for each binding mode.

#### Ethanolamine Group

**Figure 8:**
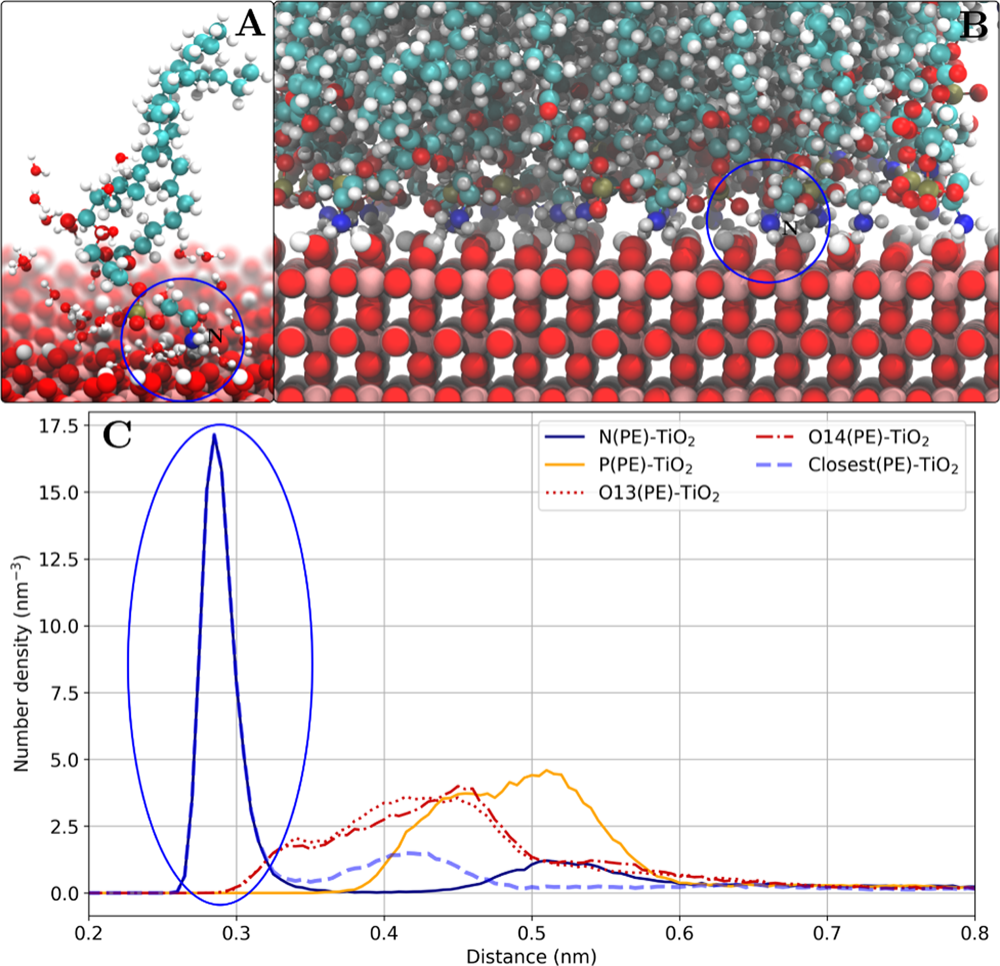
Ethanolamine binding mode as shown for the POPE lipids adsorbed on the rutile(110) surface. (A) Snapshot of one adsorbed POPE lipid (other lipid molecules are not shown) and water molecules within 0.4 nm of the POPE molecule. A direct contact of the ethanolamine group with the surface is observed. (B) Snapshot of the lipid–TiO_2_ interface. (C) Number density profile of POPE headgroup atoms. The nitrogen density peak is marked with a blue oval.

The ethanolamine group within the POPE lipid headgroup contains a positively charged, hydrogen-bond-donating fragment −NH_3_^+^. It can readily form hydrogen bonds with the oxygen bridges and hydroxyl groups on the TiO_2_ surface.^63^ Our data suggests that this binding mode is one of the main binding modes for POPE adsorption on the TiO_2_ surface. A relatively short N–TiO_2_ distance of 0.28–0.30 nm is typical for a hydrogen bond. Table 3 outlines the characteristics of the binding mode for the studied flat nanosurfaces. An example of the binding mode is shown for the POPE lipids adsorbed on the rutile(110) surface (Figure 8).

**Table 3:** POPE Binding Modes Characteristics

binding mode	TiO_2_ nanosurface	*P*_b_ (%)	residence time (ns)
phosphate (direct)	anatase(101)	1.2	250
	anatase(100)	1.3	97
ethanolamine	anatase(100)	35.1	36.6
	rutile(110)	28.5	19.9
	anatase(101)	18.2	8.1
	rutile(101)	12.1	2.1
phosphate (water-mediated)	anatase(101)	21.0	6.0
	anatase(100)	13.3	7.7
	rutile(101)	23.3	3.1

#### Phosphate Group (Direct Surface Contact)

**Figure 9:**
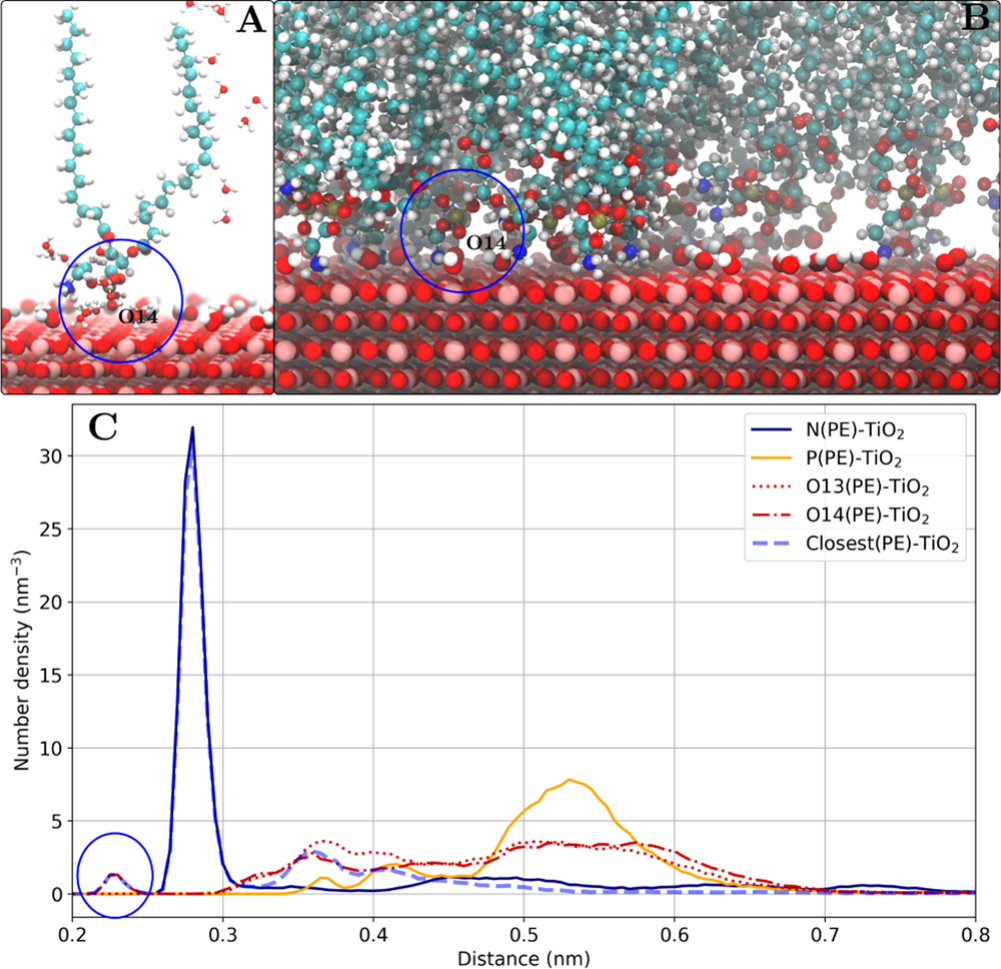
Phosphate group (direct surface contact) binding mode as shown for the POPE lipids adsorbed on the anatase(100) surface. (A) Snapshot of one adsorbed POPE lipid (other lipid molecules are not shown) and water molecules within 0.4 nm of the POPE molecule. A direct contact of the phosphate group with the surface is observed. (B) Snapshot of the lipid–TiO_2_ interface. (C) Number density profile of POPE headgroup atoms. The phosphate group oxygen (O14) density peak is marked with a blue oval.

This particular binding mode has been observed for the POPE adsorption on anatase(100) and anatase(101). Snapshots of the binding mode and the corresponding number density profile are shown in Figure 9. The number density profiles and the snapshots show that the O14 oxygen atom of the phosphate group (O14 has the most negative charge in the phosphate group along with O13) is separated by a 0.22–0.25 nm distance from the positively charge Ti atoms with no water molecules in between. Although the data in Table 3 shows that the residence time is long in comparison to the ethanolamine group binding, the overall occurrence of this type of binding is very low. The *P*_b_ value of around 1.2% with around 80 lipids in total suggests that in average only one lipid molecule in the simulated system is observed in this binding mode. A possible explanation of the low occurrence is the fact that such an adsorbed lipid has a lower configurational entropy and a higher contact surface area of the lipid headgroup.

#### Phosphate Group (Water-Mediated Interaction)

**Figure 10:**
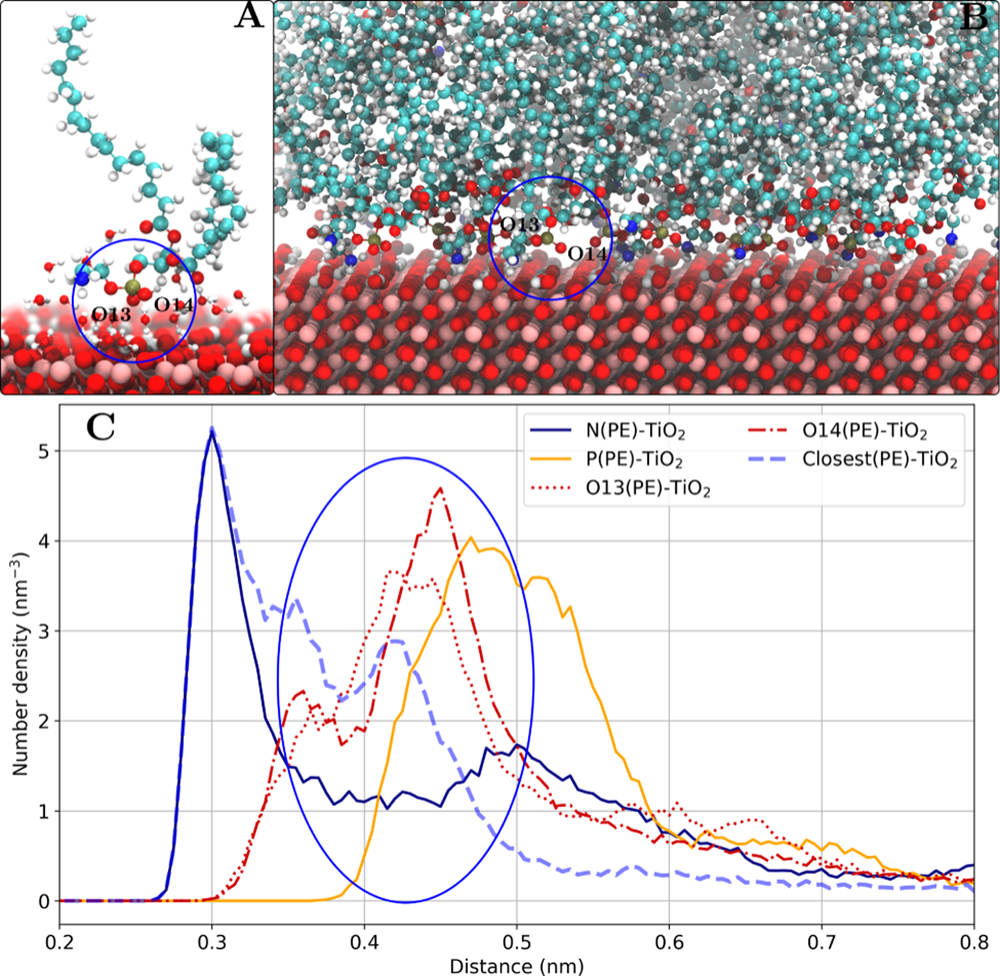
Phosphate group binding mode (water-mediated) as shown for the POPE lipids adsorbed on the rutile(101) surface. (A) Snapshot of one adsorbed POPE lipid (other lipid molecules are not shown) and water molecules within 0.4 nm of the POPE molecule. A water-mediated interaction of the phosphate group with the surface is observed. (B) Snapshot of the lipid–TiO_2_ interface. (C) Number density profile of DMPC headgroup atoms. The phosphate group oxygen (O13 and O14) density peaks are marked with a blue oval.

This type of interaction is observed for most of the combinations of both phospholipids with the studied TiO_2_ surfaces. For many of them, it is one of the few accessible binding modes. Bulkier choline group in the DMPC headgroup does not allow a close contact with the surface in most cases. The number density peaks suggest that the two most negatively charged oxygen atoms (O13 and O14) can be found near the TiO_2_ surfaces separated by 0.42–0.45 nm. A relatively large distance between the surface and the phosphate group implies that one water molecule is present between the surface and the lipid headgroup. This is further confirmed by the snapshots in Figure 10. Tables 3 and 4 show that for both POPE and DMPC lipids, the residence times are generally lower than that for ethanolamine or direct phosphate group binding. However, the fraction of lipids adsorbed via this binding mode is on par with the ethanolamine group binding.

**Table 4:** DMPC Binding Modes Characteristics

Binding mode	TiO_2_ nanosurface	*P*_b_ (%)	residence time (ns)
glycerol moiety	anatase(101)	1.2	500.1
choline (direct)	rutile(101)	15.7	3.5
phosphate (water-mediated)	anatase(101)	13.4	5.8
	rutile(110)	8.5	3.9
	rutile(101)	5.2	1.0
	anatase(100)	1.6	1.8
choline (water-mediated)	rutile(110)	10.7	1.3
	rutile(101)	8.2	1.2
	anatase(101)	7.9	1.3
	anatase(100)	2.4	0.9

#### Glycerol Moiety

**Figure 11:**
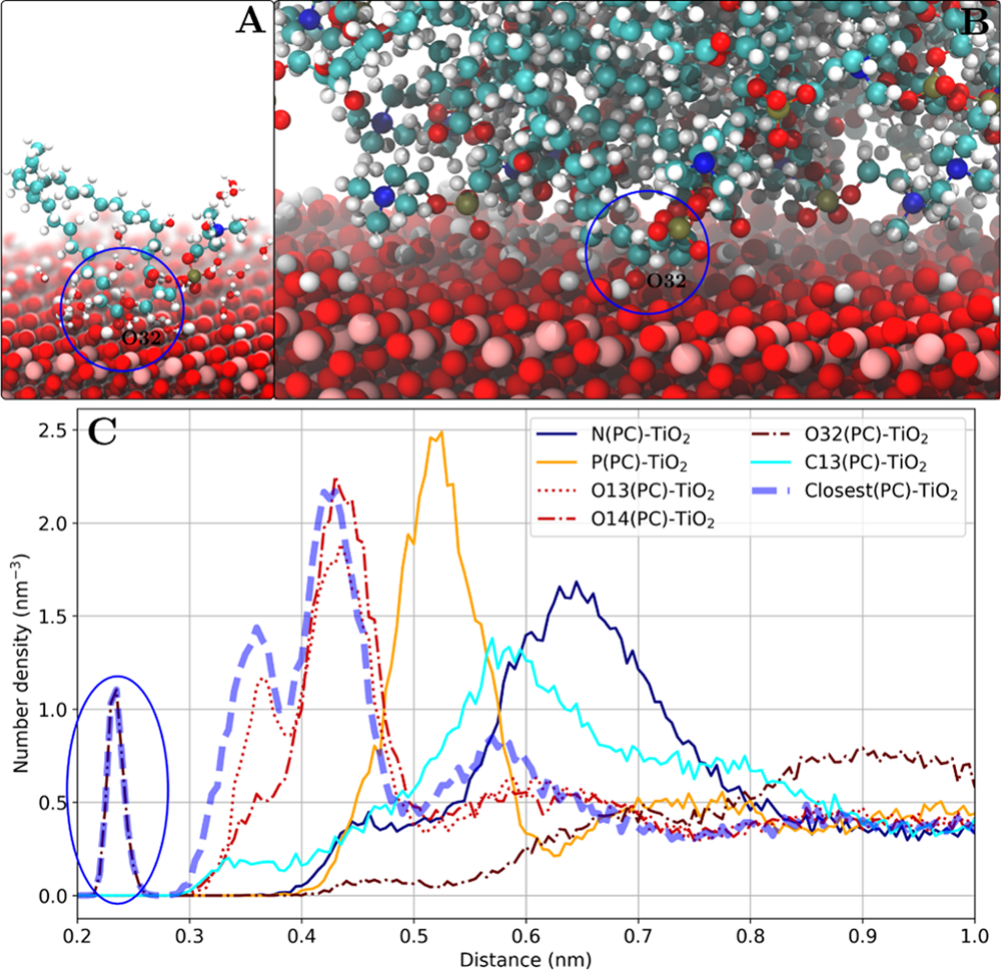
Glycerol moiety binding mode through a carbonyl oxygen atom (O32) as shown for the DMPC lipids adsorbed on the anatase(101) surface. (A) Snapshot of one adsorbed DMPC lipid (other lipid molecules are not shown) and water molecules within 0.4 nm of the DMPC molecule. A direct contact of the carbonyl oxygen of the glycerol moiety (O32) with the surface is observed. (B) Snapshot of the lipid–TiO_2_ interface. (C) Number density profile of DMPC headgroup atoms. The carbonyl oxygen (O32) density peak is marked with a blue oval.

It was found that in the case of DMPC adsorption on the anatase(101) surface, the carbonyl oxygen in the glycerol moiety (O32) can approach the surface directly with separations of around 0.22–0.25 nm. The snapshots of the system (Figure 11) suggest that a hydroxyl group on the surface mediates the interaction. However, no unbound water is found between the adsorbed lipid and the TiO_2_ surface. Table 4 reveals the similarities between the glycerol moiety binding mode and the direct phosphate group binding—both have very low occurrence (only one such lipid is observed on average during the simulation), very short characteristic separation (around 0.22–0.25 nm from the surface), and very long residence time (at least hundreds of nanoseconds) compared to other, more common binding modes.

#### Choline Group (Direct Surface Contact)

**Figure 12:**
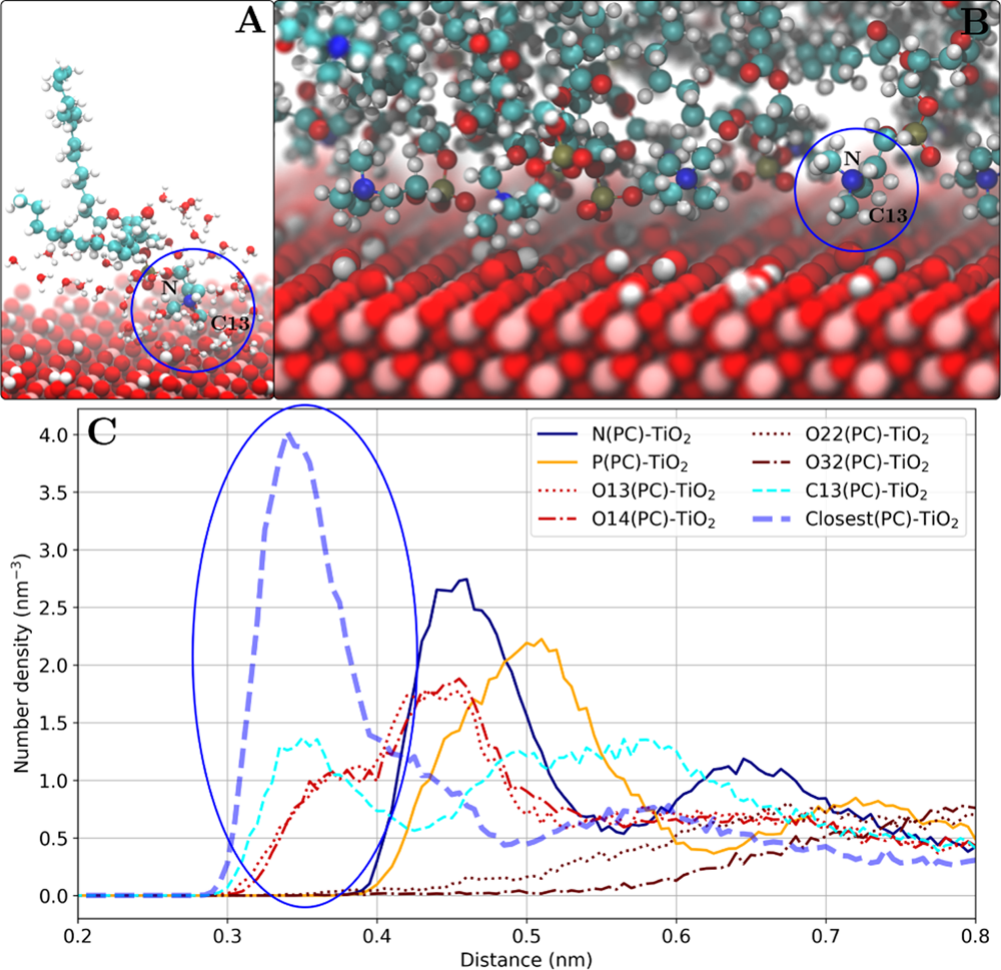
Choline group (direct surface contact) binding mode as shown for the DMPC lipids adsorbed on rutile(101) surface. (A) Snapshot of one adsorbed DMPC lipid (other lipid molecules are not shown) and water molecules within 0.4 nm of the DMPC molecule. A direct contact of the choline group with the surface is observed. (B) Snapshot of the lipid–TiO_2_ interface. (C) Number density profile of DMPC headgroup atoms. The choline group carbon atom (C13, C14, and C15 are equivalent) density peak is marked with a blue oval.

In the case of DMPC adsorption on rutile(101), a direct contact of the choline group with the surface is often observed. A snapshot of direct choline group binding and a corresponding number density profile is shown in Figure 12. Table 4 shows the calculated binding mode characteristics. It seems that a relatively high surface charge density of rutile(101) surface in our simulations has brought the choline groups very close to the surface with the choline carbon atom in the range of 0.3–0.4 nm from the surface and with a residence time of around 3.5 ns. A short separation suggests no water molecules between the choline group and the TiO_2_ surface. This is the only case for DMPC adsorption where we have found choline groups closer than the phosphate groups.

#### Choline Group (Water-Mediated Interaction)

**Figure 13:**
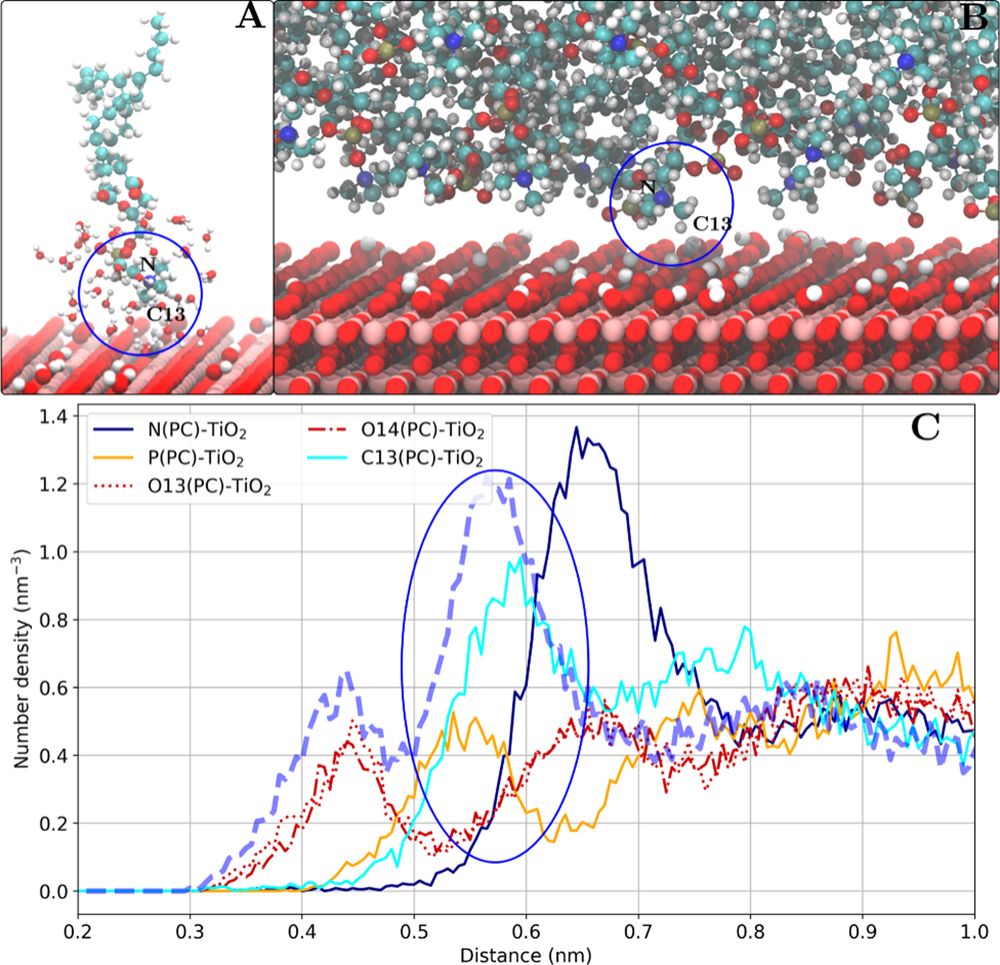
Choline group (water-mediated) binding mode as shown for the DMPC lipids adsorbed on rutile(110) surface. (A) Snapshot of one adsorbed DMPC lipid (other lipid molecules are not shown) and water molecules within 0.4 nm of the DMPC molecule. A water-mediated interaction of the choline group with the surface is observed. (B) Snapshot of the lipid–TiO_2_ interface. (C) Number density profile of DMPC headgroup atoms. The choline group carbon atom (C13, C14, and C15 are equivalent) density peak is marked with a blue oval.

In the rest of the investigated systems, choline groups interact with the TiO_2_ surface only through a layer of water molecules as seen in Figure 13. Table 4 suggests that the water-mediated interactions are weaker compared to the direct choline group binding with the lower fractions of adsorbed lipids as well as lower residence times. Notably, a small number density peak, corresponding to the water-mediated choline group interaction is observed in rutile(101) along with the direct contact.

#### Effect of Cholesterol

**Figure 14:**
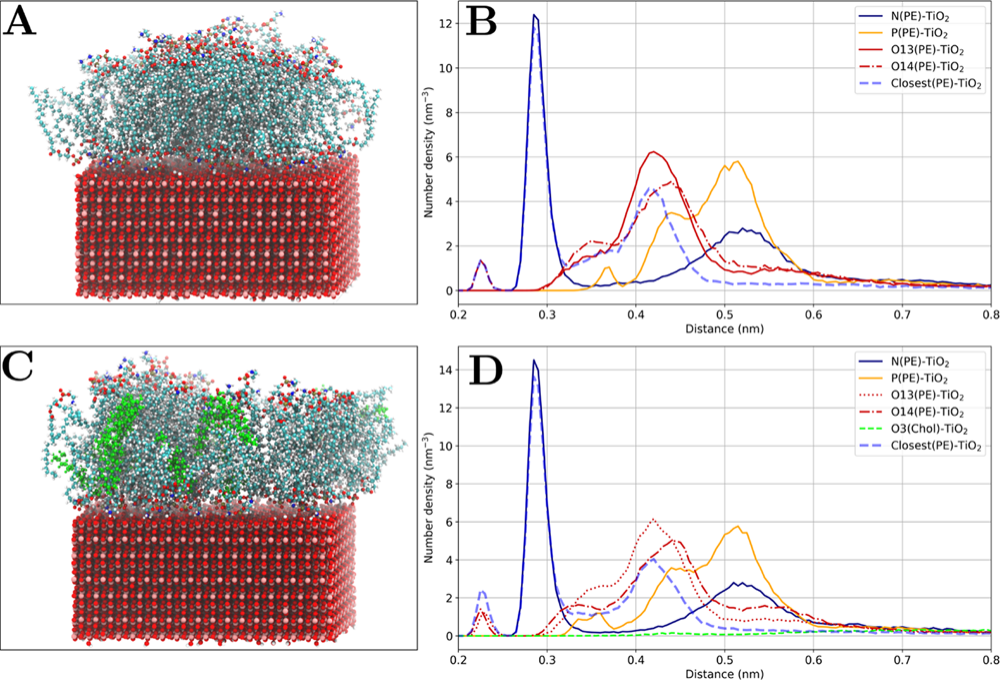
Effect of cholesterol on POPE adsorption. (A) Snapshot of 82 POPE on anatase(101). (B) Corresponding number density profile for POPE lipids. (C) Snapshot of 82 POPE and 16 cholesterol (shown in light green) molecules on anatase(101). (D) Corresponding number density profile for POPE lipids and cholesterol.

**Figure 15:**
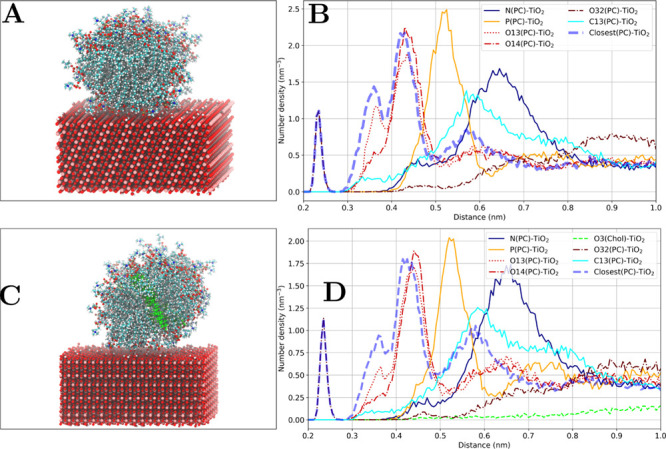
Effect of cholesterol on DMPC adsorption. (A) Snapshot of 82 DMPC on anatase(101). (B) Corresponding number density profile for DMPC lipids. (C) Snapshot of 82 DMPC and 16 cholesterol (shown in light green) molecules on anatase(101). (D) Corresponding number density profile for DMPC lipids and cholesterol.

Simulations of the anatase(101) slab with mixtures of POPE and DMPC lipids with cholesterol have revealed its effect on the phospholipid adsorption. Figures 14 and 15 show snapshots and comparison of the number density profiles for pure phospholipids and their mixtures with cholesterol. The number density profiles show that cholesterol does not attach to the surface directly and the number density of the hydroxyl group oxygen atom (O3) in cholesterol is much lower in comparison to the number density of the phospholipid atoms, even considering the 1:5 concentration proportionality. Thus, cholesterol molecules mostly stay in the nonpolar part of the lipid bilayers. However, Table 5 shows a minor effect of cholesterol on the binding mode characteristics: ethanolamine binding in POPE and choline binding in DMPC becomes slightly more favored compared to adsorption of pure phospholipids. This may be attributed to a well-known effect of decreasing the area per lipid in phospholipid–cholesterol mixtures.^64,65^ As in the case of increased lipid concentration, binding of ethanolamine and choline groups is favored due to the smaller contact surface area compared to the phosphate group binding.

**Table 5:** Effect of Cholesterol on the Binding Mode Characteristics

	POPE		POPE + cholesterol	
binding mode	*P*_b_ (%)	residence time (ns)	*P*_b_ (%)	residence time (ns)
phosphate group (direct)	1.2	250	2.4	250
ethanolamine group	18.2	8.1	21.4	10.1
phosphate group (water-mediated)	21.0	6.0	18.7	5.5

	DMPC		DMPC + cholesterol	
binding mode	*P*_b_ (%)	residence time (ns)	*P*_b_ (%)	residence time (ns)
glycerol moiety	1.2	500	1.2	500
phosphate group (water-mediated)	13.4	5.8	10.7	3.9
choline group (water-mediated)	7.9	1.3	9.3	1.3

#### Effect of TiO_2_ Surface Curvature

**Figure 16:**
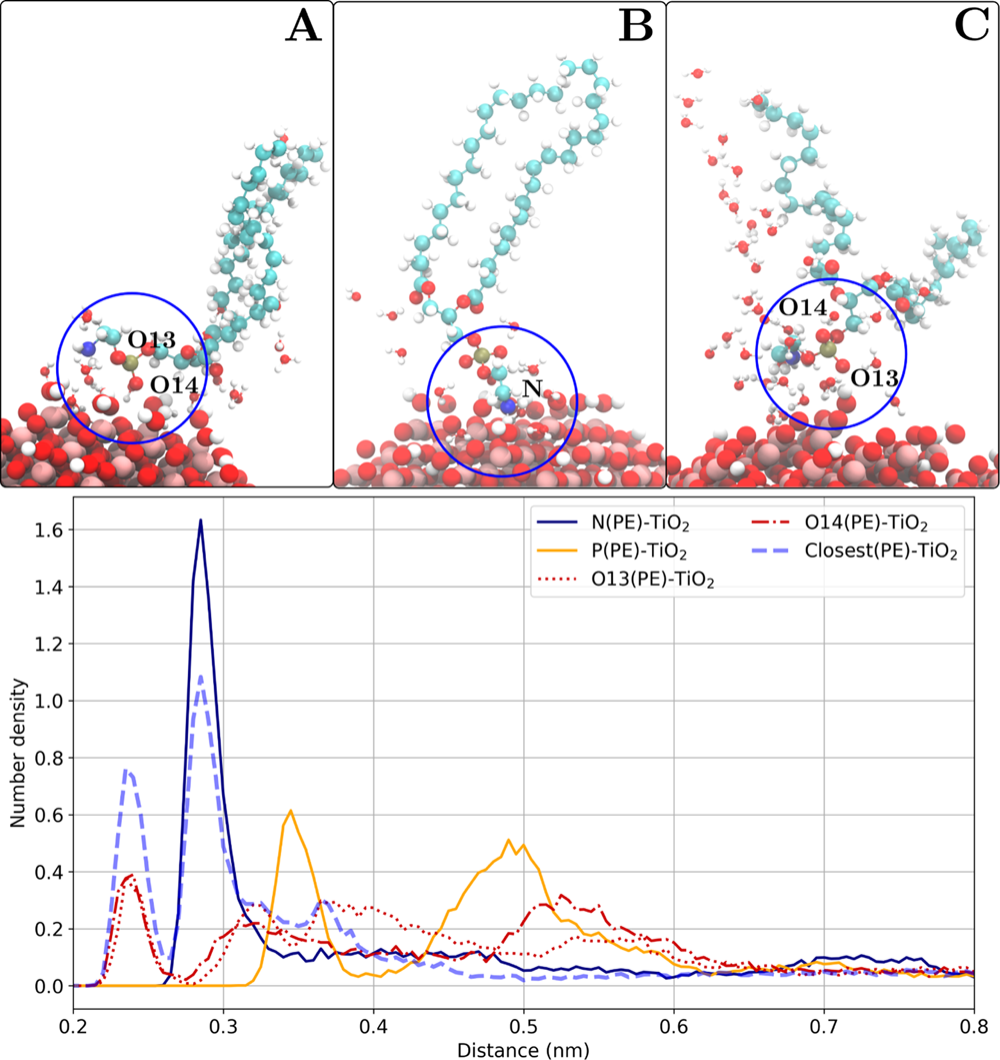
Anatase nanoparticle–POPE lipid headgroup atoms number density profiles and the corresponding representations of the binding modes. (A) Phosphate group (direct contact), (B) ethanolamine group, and (C) phosphate group (water-mediated).

Simulation of anatase nanoparticle with POPE lipids shows that the binding modes appear to be similar to those that are found for POPE adsorption on the anatase(100) and anatase(101) surfaces as shown in Figure 16. However, their characteristics differ significantly. Table 6 shows the comparison between POPE adsorption on flat surfaces and the anatase nanoparticle. Arguably, the differences might be explained by the fact that the curved geometry of the nanoparticle allows for more flexible lipid configurations compared to the flat surfaces. Additionally, a higher concentration of hydroxyl groups and other surface defects affect the way lipid molecules interact with TiO_2_ surface.

**Table 6:** Comparison between POPE Adsorption on Flat and Curved Surfaces

	anatase(101)		anatase(100)		anatase nanoparticle (*r* = 2 nm)	
binding mode	*P*_b_ (%)	residence time (ns)	*P*_b_ (%)	residence time (ns)	*P*_b_ (%)	residence time (ns)
phosphate group (direct)	1.2	249.9	1.3	97.1	4.3	15.0
ethanolamine group	18.2	8.1	35.1	36.6	7.2	4.1
phosphate group (water-mediated)	21.0	6.0	13.3	7.7	5.8	3.2

#### Effect of Lipid Concentration and Simulation Length

Additional analysis of the simulations carried out for the anatase(101) surface with increased number of lipids and with extended 5 μs simulation time is presented in Supporting Information Figures S8–S10 and Tables S3–S4, respectively. These data essentially confirm the results described in the sections above.

## Discussion

Using the calculated number density profiles of all of the studied systems, one can estimate the total fraction of adsorbed lipids by assuming a certain adsorption layer width. One can define the adsorption layer from the TiO_2_ surface (0 nm separation) up to 1 nm. It corresponds to an approximate thickness of the headgroup region in a lipid bilayer and, at least in the case of the POPE bilayer adsorption, the number density of the closest headgroup atoms goes very close to zero at 1 nm (see Figures S6 and S7). Then, the calculated fraction of the adsorbed lipids can be related to the overall adsorption strength. The fractions of adsorbed lipids on TiO_2_ nanosurfaces are shown in Table 7. For both anatase and rutile surfaces, one can immediately see that the adsorption of POPE lipids is generally stronger than for DMPC lipids. Moreover, within each type of lipids, there is a certain preference toward specific TiO_2_ surfaces. For POPE lipids, the adsorption increases in the following sequence: anatase (NP) ≪ rutile(101) ≈ rutile(110) < anatase(101) < anatase(100). For DMPC lipids, we see a different trend in relative adsorption strength: anatase(100) ≪ rutile(110) < anatase(101) < rutile(101). Interestingly, while the adsorption of POPE lipids on anatase(100) is the strongest, for DMPC lipids, it is the weakest. The opposite is true for the rutile(101) surface. Strong adsorption of DMPC lipids on the rutile(101) surface was observed in a recent simulation study that used the same force field for DMPC and titania.^29^ However, probably due to the lack of charged groups on the surface in the aforementioned study, there was no significant difference between DMPC adsorption on anatase(101) or anatase(100) as we observe in our simulations.

**Table 7:** Comparing the Total Fraction of Adsorbed Lipids for TiO_2_ Nanosurfaces

TiO_2_ surface	*N*_POPE_	fraction of adsorbed lipids (%)	adsorption layer width (nm)
anatase(100)	76		1
anatase(101)	120	50.0	1
anatase(101)^b^	82	49.0	1
anatase(101)^c^	82	48.2	1
anatase(101)^a^	82	46.5	1
anatase NP	83	22.8	1
rutile(110)	85	42.9	1
rutile(101)	82	42.9	1

TiO_2_ surface	*N*_DMPC_	fraction of adsorbed lipids (%)	adsorption layer width (nm)
anatase(101)	120	38.0	1
anatase(101)	82	28.4	1
anatase(101)^c^	82	27.3	1
anatase(100)	76	5.0	1
rutile(101)	82	34.3	1
rutile(110)	85	22.2	1

a 1 μs simulation.

b 5 μs simulation.

c Additional 16 cholesterol molecules.

The fraction of adsorbed lipids (both for POPE and DMPC) does not change significantly with an addition of cholesterol in 1:5 proportion to the anatase(101) system. An additional 5 μs simulation shows that the fraction of adsorbed POPE lipids on the anatase(101) surface has changed by around 10% compared to the 1 μs simulation. This may suggest that longer sampling is necessary for the TiO_2_–lipid systems.

Our simulations of TiO_2_ slabs were carried out for ideal plain surfaces. Real TiO_2_ nanosurfaces can have various defects depending on the synthesis and other conditions, which can affect binding of biomolecules. Our simulation of a spherical anatase nanoparticle showed generally weaker binding of lipids compared to binding to anatase plain surfaces, which can be a combination of effects of curvature and surface defects. More detailed investigations of how different defects at TiO_2_ surfaces as well as eventual functionalization affect binding can be a matter of further studies.

## Conclusions

We have performed all-atom molecular dynamics simulations of DMPC and POPE lipids near various low-energy anatase and rutile surfaces and at spherical nanoparticle. Both lipid molecules, initially dispersed in water, spontaneously form aggregates and adsorb on TiO_2_ surfaces.

By calculating the number density profiles for lipid headgroup atoms, we have identified several TiO_2_–lipid binding modes. The residence times were estimated for each binding mode. We have found that the binding modes with the highest retention are the phosphate in direct contact with the surface as well as carbonyl oxygen in DMPC. These results are in line with the findings from previous simulation study of lipids near TiO_2_ surfaces.^42^ However, the aforementioned binding modes are very rare in comparison to the direct ethanolamine group binding in POPE and water-mediated interaction of the phosphate with the surface found in both POPE and DMPC. Choline group binding in DMPC has relatively high characteristic separations from the surface and short residence times, as expected from its less polar structure in comparison to the ethanolamine, although it is found to be one of the main binding modes in the DMPC adsorption on the rutile(101) surface. Additional simulations of anatase(101) with cholesterol and the phospholipids in 1:5 mixture have shown a minor effect of cholesterol on the phospholipid adsorption. While cholesterol does not interact with the surface directly as it stays in the nonpolar part of the lipid aggregate, the systems with cholesterol have shown a slightly higher preference toward ethanolamine group binding in POPE and choline group binding in DMPC. Simulation of POPE lipids near a small anatase NP (2 nm) has revealed that while the number density profiles and the binding modes are similar to those obtained for flat anatase surfaces, the peak areas are much smaller, the residence times of lipids are shorter, and the overall adsorption is weaker than anatase(101) or anatase(100).

Integrating the normalized number density peaks has allowed us to estimate the total fraction of adsorbed lipids for different systems during the simulations and thus arguing about the relative strength of lipid adsorption on various TiO_2_ surfaces. Our data suggests that POPE lipid adsorption is generally stronger than DMPC adsorption. This may be attributed to the fact that choline group binding is not as strong as the ethanolamine group binding. Furthermore, we have observed a stronger adsorption for POPE lipids on anatase than on rutile. For DMPC, the trend is different—adsorption on rutile(101) is found to be the strongest, which is in agreement with another simulation study.^29^ After rutile(101), adsorption decreases in the sequence of anatase(101), rutile(110), and anatase(100). Adsorption on anatase(100) is found to be considerably weaker than for other surfaces. The present work provides a detailed study of the differences in the adsorption of phospholipids on various TiO_2_ surfaces as well as a methodology for quantifying the adsorption of biomolecules on inorganic surfaces.

## Supporting Information Available

Nonbonded force-field parameters (Table S1); bonded force-field parameters for TiO_2_ (Table S2); snapshots of different anatase(100) and anatase(101)–lipid systems (Figure S1); snapshots of different rutile(101) and rutile(110)–lipid systems (Figure S2); radial distribution functions (Figure S3); number density profiles (Figure S4–S7); comparison between full and partial bilayer adsorption on anatase(101) (Table S3); comparison of adsorption of partial and full POPE lipid bilayers (Figure S8); comparison between full and partial DMPC lipid bilayers (Figure S9); comparison between 1 and 5 μs long simulations of anatase(101) with POPE (Figure S10); and comparison of binding mode characteristics for 1 and 5 μs long simulations of anatase(101) with POPE (Table S4) (PDF)
Molecular dynamics setup and topology files with force field parameters for all nanosurfaces and molecules used in the simulations, in Gromacs format (ZIP)
