# Supplementary material for: Atomistic Molecular Dynamics Simulations of Lipids Near TiO2 Nanosurfaces
Source: J Phys Chem B. 2021 Jul 16;125(29):8048–59. doi: 10.1021/acs.jpcb.1c04547 (PMC8389913; doi:10.1021/acs.jpcb.1c04547)
Supplement: Supplementary file 1 — jp1c04547_si_001.pdf [file jp1c04547_si_001.pdf]

Atomistic Molecular Dynamics Simulations of Lipids Near TiO_2 Nanosurfaces Supporting Information

Mikhail Ivanov and Alexander P. Lyubartsev*

Department of Materials and Environmental Chemistry, Stockholm University, SE-106 91, Stockholm, Sweden

E-mail: alexander.lyubartsev@mmk.su.se

Phone: +46 8 161193

Force field parameters for titania

Table S1: Non-bonded force field parameters for TiO_2. For each atom type, coordinated atoms are given in parenthesis

Atom type	Comment	$q(e)$	σ(Å)	ϵ(kJ/mol)
Ti(O6)	Bulk Ti	2.248	1.9	13.79
Ti(O5)	Surface Ti	2.159	1.9	13.79
O(Ti3)	Oxygen in TiO_2 bulk	-1.124	3.51	0.409
O(Ti2)	Bridge oxygen on TiO_2	-1.035	3.42	0.401
O(Ti,H)	Hydroxyl oxygen	-0.913	3.29	0.389
H(O)	Hydrogen	0.417	0	0

Table S2: Bonded force field parameters for TiO_2

Bond type	b_o(Å)	k_b (kJ/mol Å^2)
Ti-O(Ti3) bulk	1.9	8000.
Ti-O(Ti2) bridge	1.9	8000.
Ti-O(H) hydroxyl	1.9	8000.
O-H hydroxyl	1.0	3267.
Angle type	θ_0 (deg)	k_θ (kJ/mol deg^2)
Ti-O-H hydroxyl	114.85	5433.

System snapshots

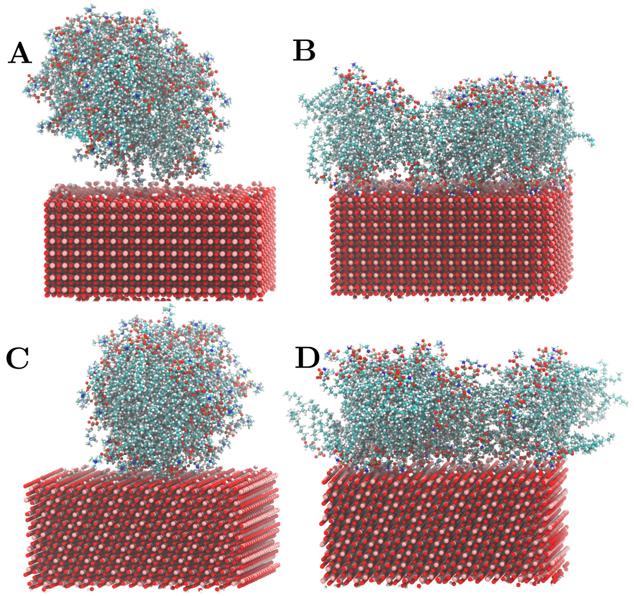

Figure S1: Snaphost of different anatase (100) and anatase (101) - lipid systems. A - anatase (100) - DMPC; B - anatase (100) - POPE; C - anatase (101) - DMPC; D - anatase (101) - POPE.

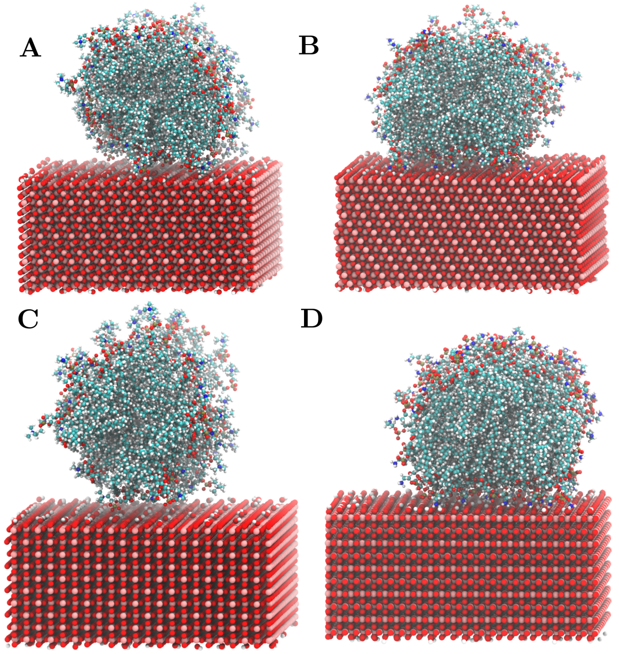

Figure S2: Snapshots of different rutile (101) and rutile (110) - lipid systems. A - rutile (101) - DMPC; B - rutile (101) - POPE; C - rutile (110) - DMPC; D - rutile (110) - POPE.

Radial distribution functions

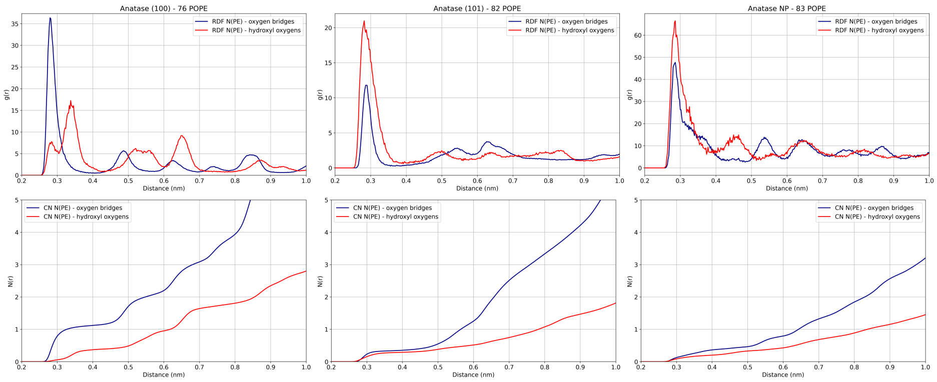

Figure S3: Radial distribution functions N - TiO_2 (oxygen bridge) and N - TiO_2 (hydroxyl oxygen), as well as the corresponding coordination numbers for anatase (100), anatase (101) and anatase NP in contact with POPE.

Additional number density profiles

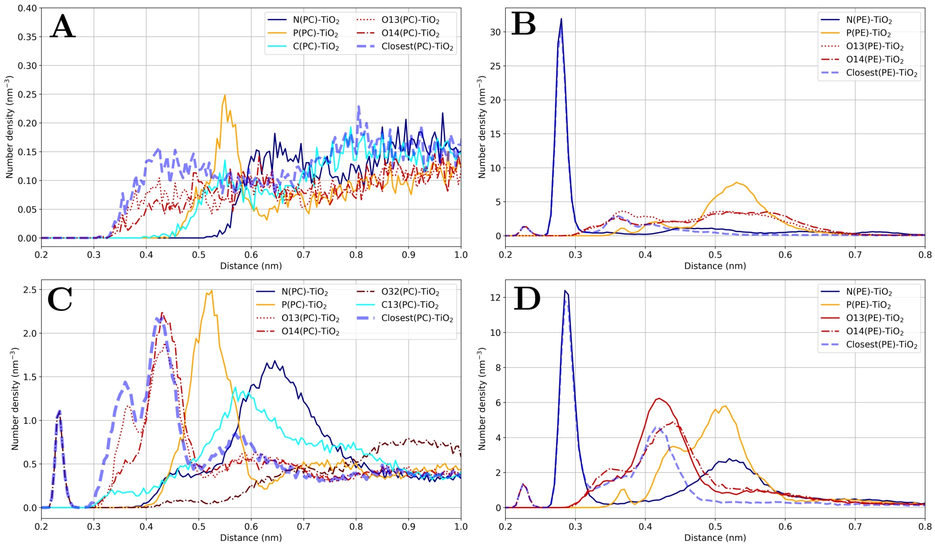

Figure S4: Number density profiles for different anatase (100) and anatase (101) - lipid systems. A - anatase (100) - DMPC; B - anatase (100) - POPE; C - anatase (101) - DMPC; D - anatase (101) - POPE.

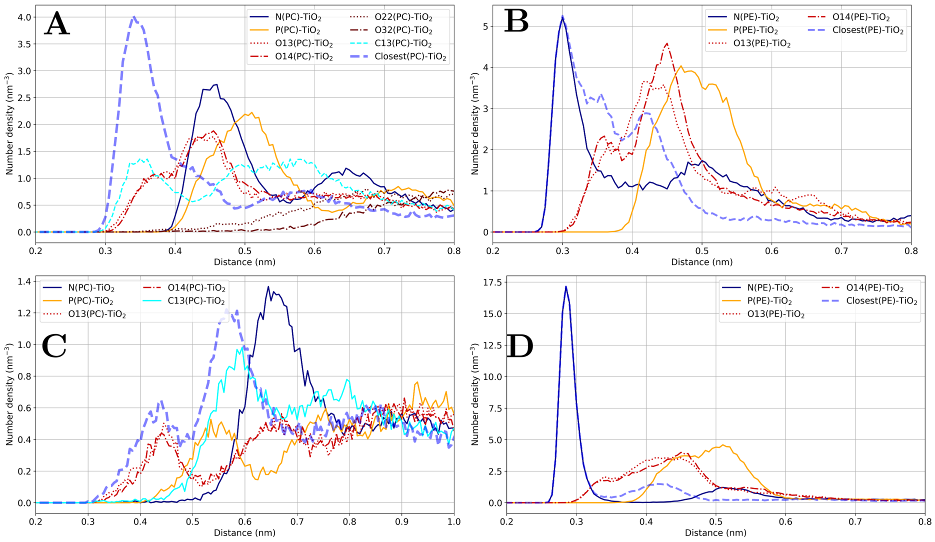

Figure S5: Number density profiles for different rutile (101) and rutile (110) - lipid systems. A - rutile (101) - DMPC; B - rutile (101) - POPE; C - rutile (110) - DMPC; D - rutile (110) - POPE.

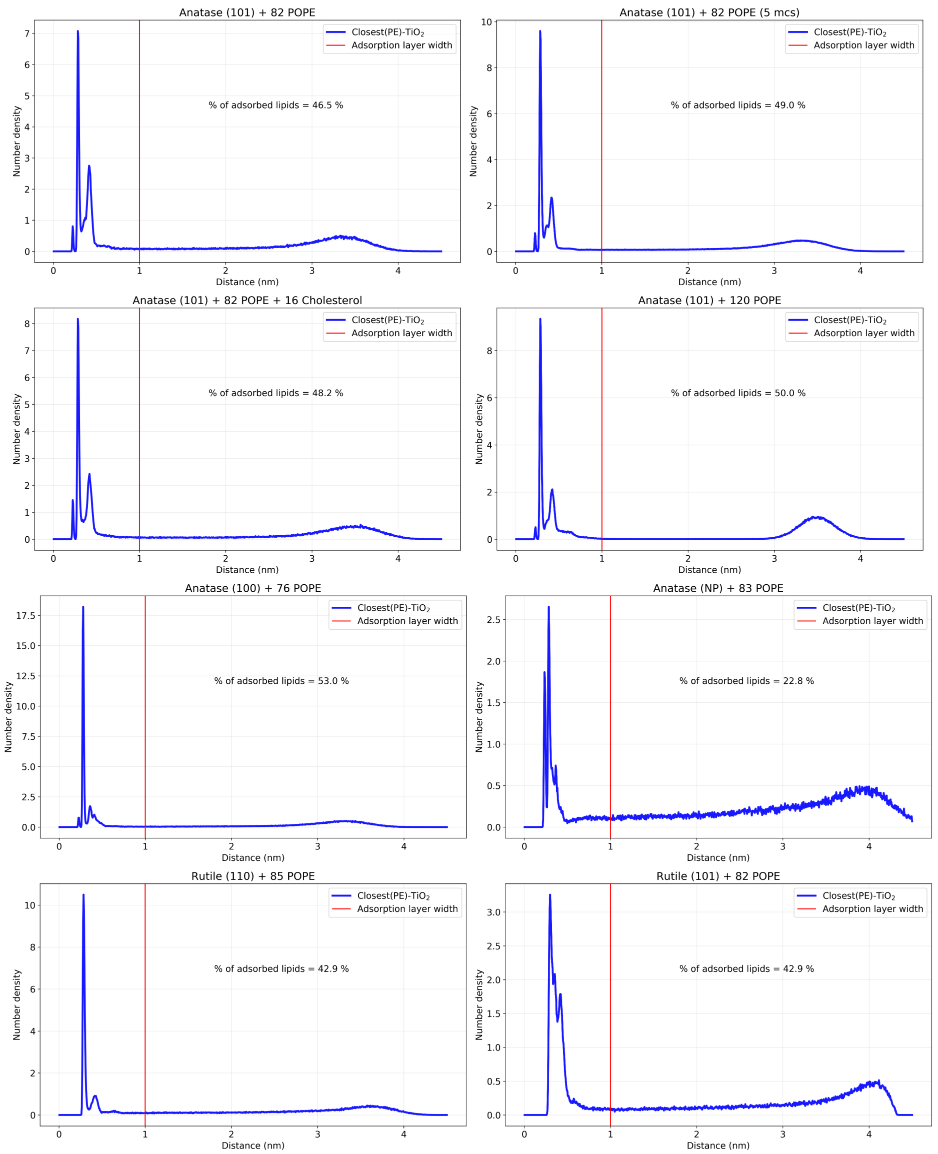

Figure S6: Number density profiles for different TiO_2 surfaces in contact with POPE in a large range of distances.

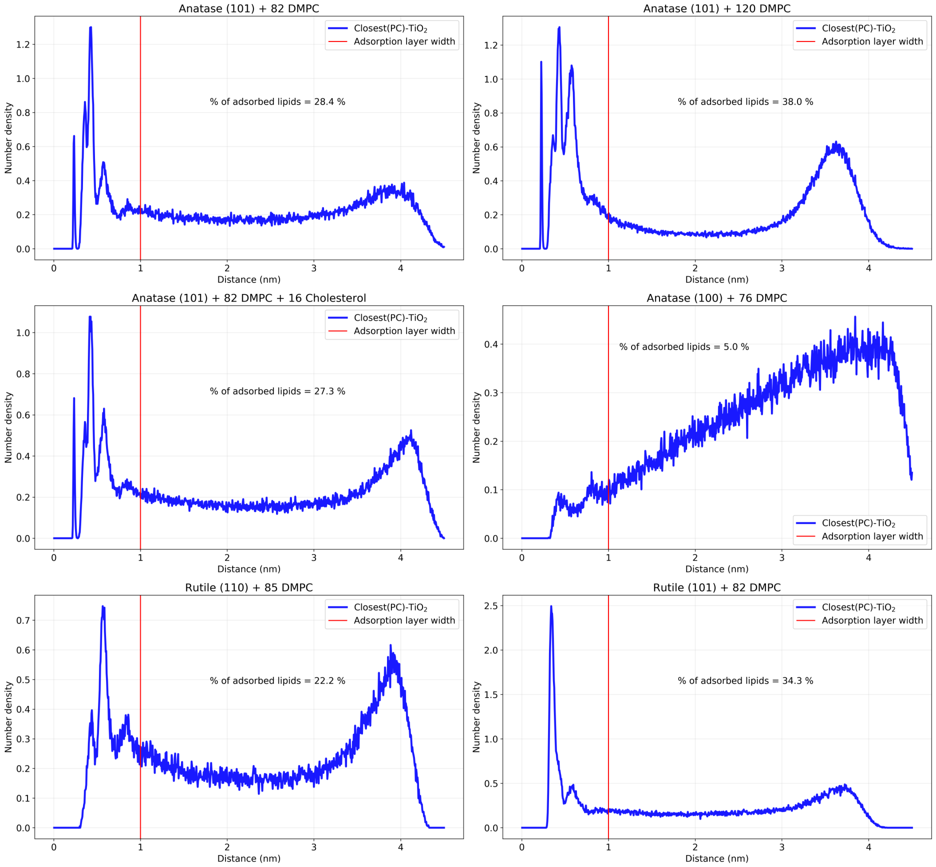

Figure S7: Number density profiles for different TiO_2 surfaces in contact with DMPC in a large range of distances.

Effect of lipid concentration

To study the effect of the lipid concentration on the their adsorption on TiO_2 surfaces, two additional simulations were carried out - anatase (101) slab with 120 DMPC and with 120 POPE lipids. In these two simulations, the total number of lipids is roughly 50% higher than in other systems, and the lipids almost form a complete bilayer. The snapshots of the systems with both higher and lower concentration of lipid molecules and the corresponding number density profiles are shown in Figures S8-S9. Although the lipid packing changes with increasing concentration, the number density profiles remain very similar - their position is the same and only the peak area has changed. The binding mode characteristics comparison is presented in Table S3. Our data suggests that the effect of increasing lipid concentration is similar for both POPE and DMPC: ethanolamine and choline group binding becomes more pronounced. One can argue that with denser packing in bilayers, binding through the end portion of the headgroup (ethanolamine in POPE and choline in DMPC) is more favorable as the contact area of the headgroup and the surface is lower in that case.

Table S3: Comparison between full and partial bilayer adsorption on anatase (101)

POPE				
Binding mode	Partial bilayer		Full bilayer	
	P_b, %	Residence time, ns	P_b, %	Residence time, ns
Phosphate group (direct)	1.2	249.9	0.8	316.6
Ethanolamine group	18.2	8.1	23.6	12.7
Phosphate group (water-mediated)	21.0	6.0	17.4	4.8
DMPC				
Binding mode	Partial bilayer		Full bilayer	
	P_b, %	Residence time, ns	P_b, %	Residence time, ns
Glycerol moeity	1.2	500.	1.7	667
Phosphate group (water-mediated)	13.4	5.8	13.8	3.2
Choline group (water-mediated)	7.9	1.3	15.8	1.8

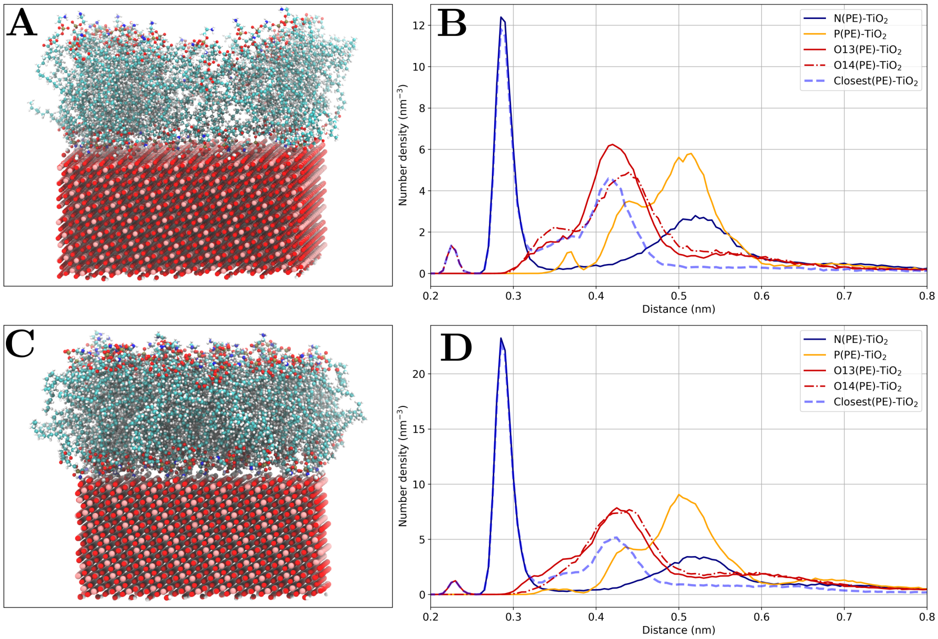

Figure S8: Comparison of adsorption of partial and full POPE lipid bilayer. A - A snapshot of 82 POPE on anatase (101); B - A corresponding number density profile for the partial bilayer; C - A snapshot of 120 POPE on anatase (101); D - A corresponding number density profile for the full bilayer.

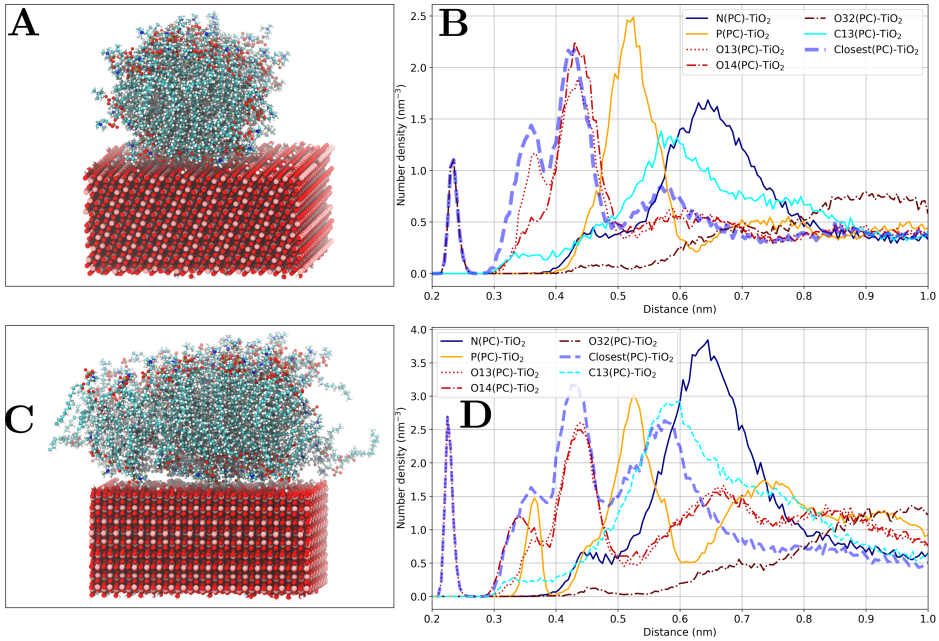

Figure S9: Comparison between full and partial DMPC lipid bilayer. A - A snapshot of 82 DMPC on anatase (101); B - A corresponding number density profile for the partial bilayer; C - A snapshot of 120 DMPC on anatase (101); D - A corresponding number density profile for the full bilayer.

Effect of simulation length

Figure S10 shows the similarities between the obtained results for 1 and 5 μs simulations of anatase (101) and POPE system. However, a closer analysis of the longer trajectory reveals that there are certain quantitative differences between 1 and 5 μs simulations. The data is shown in Table S4. In the longer simulation, more POPE lipids are observed to be adsorbed through the ethanolamine group as well as less lipids are absorbed through the phosphate group. Additionally, the estimated residence times are larger for the 5 μs simulation. However, this may be attributed to the fact that the simulation is itself longer. The data suggests that longer simulations or enhanced sampling methods might be beneficial for obtaining more accurate results on lipid adsorption on TiO_2 surfaces.

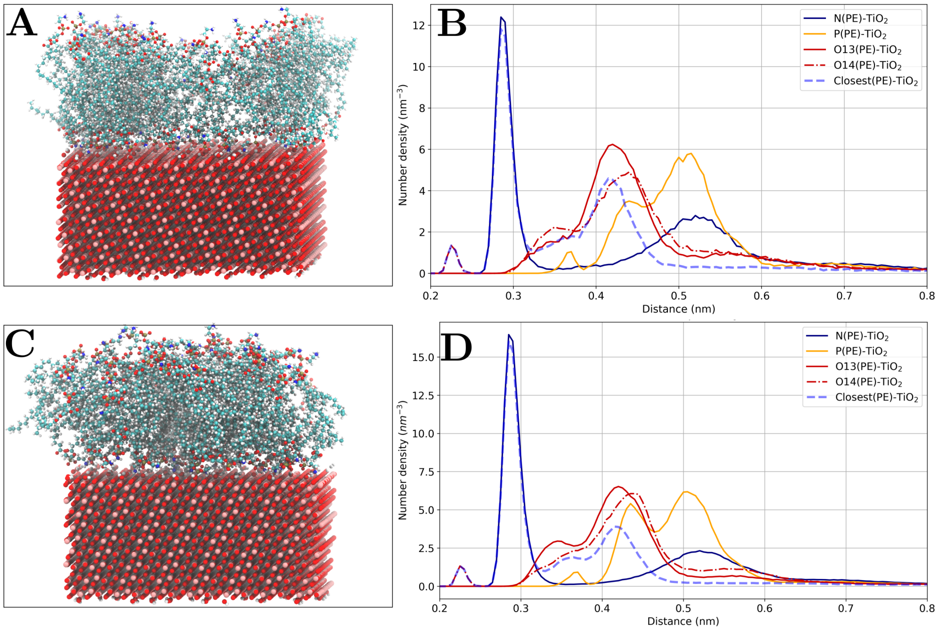

Figure S10: Comparison between 1 and 5 μs long simulations of anatase (101) with POPE. A - A snapshot of the system after 1 μs; B - A corresponding number density profile for the 1 μs long simulation; C - A snapshot of the system after 5 μs; D - A corresponding number density profile for the 5 μs simulation.

Table S4: Comparing binding mode characteristics for 1 and 5 μs long simulations of anatase (101) with POPE

Binding mode	1 μs		5 μs	
	$P_b, \%$	Residence time, ns	$P_b, \%$	Residence time, ns
Phosphate group (direct)	1.2	250	1.2	1250
Ethanolamine group	18.2	8.1	24.4	12.9
Phosphate group (water-mediated)	21.0	6.0	18.6	6.5
